# Overcoming Barriers in the Management of Hypertension: The Experience of the Cardiovascular Health Program in Chilean Primary Health Care Centers

**DOI:** 10.1155/2012/405892

**Published:** 2012-06-04

**Authors:** Daniela Sandoval, Miguel Bravo, Elard Koch, Sebastián Gatica, Ivonne Ahlers, Oscar Henríquez, Tomás Romero

**Affiliations:** ^1^Department of Family Medicine & Primary Care, Faculty of Medicine, University of Chile, 8380453 Santiago, Chile; ^2^Institute of Molecular Epidemiology (MELISA), Faculty of Medicine, Universidad Católica de la Santísima Concepción, 4070129 Concepción, Chile; ^3^School of Medicine, University of California, San Diego, CA 92093, USA; ^4^Fundacion Araucaria Foundation, Coronado, CA 92118, USA

## Abstract

*Objective*. To assess the blood pressure control and cardiovascular risk factors (CVRFs) in a population of hypertensive patients with access to care under a government-financed program, the Cardiovascular Health Program (CHP). *Design*. A cross-sectional and multicenter study. *Setting*. 52 primary care centers, metropolitan area of Santiago, Chile. *Participants*. 1,194 patients were selected by a systematic random sampling from a universe of 316,654 hypertensive patients. *Key Measurements*. Demographic information, blood pressure (BP) measurements, and CVRF were extracted from medical records of patients followed for a 12-month period. *Results*. 59.7% of patients reached target BP <140/90 mmHg. More women were captured in the sampling (2.1 : 1), achieving better BP control than men. Diabetic patients (26.4%) had worse BP control than nondiabetics. Antihypertensive medications were used in 91.5%, with multidrug therapy more frequent in patients with higher BP and more difficult control. *Conclusions*. The success in improving the BP control to values <140/90 mmHg from 45.3% to 59.7% underscores the contribution of this program in the Chilean primary care cardiovascular preventive strategies. However, fewer hypertensive men than women were captured by this program, and it is of concern the underperforming of BP control observed in diabetics.

## 1. Introduction

Hypertension control has been instrumental to achieve a significant reduction in cardiovascular events. However, despite the advancements in antihypertensive drug therapy, success in controlling BP to the Joint National Committee VII guidelines (BP < 140/90 mmHg) has been quite disappointing. Most of the published information has reported satisfactory BP control in only 30% to 45% of the hypertensive population [1–5].

Numerous factors have been mentioned as responsible for this limited results including socioeconomic status, barriers in the access to health care, lack of a population-oriented program focused on preventive cardiovascular measures, poor adherence, among others [1, 6–8]. The Chilean Ministry of Health started a Cardiovascular Health Program (CHP) (Programa de Salud Cardiovascular, PSCV) in 2002 with the objective of reducing the cardiovascular morbidity and mortality by a global management strategy of cardiovascular risk factors (CVRF) in patients followed at the primary care level [9, 10]. Approximately 76.9% of the Chilean population is enrolled in a primary care clinic through the public health system. This is financed by the Ministry of Health through subsidies to the primary care centers and clinics, some of them belonging to the private sector [9]. Patients have access to the CHP once a diagnosis of hypertension, diabetes, and/or dyslipidemia is established after the initial primary care evaluation. By law every patient enrolled in the public health system is eligible for a preventive annual medical examination. Patients referred to the CHP receive free followup and drug therapy at least every three months through multidisciplinary teams composed by primary care physicians, nurses, dietitians, and social workers. Patients are referred to a secondary health care system when secondary hypertension is suspected or when they develop complications such as ischemic heart disease, congestive heart failure, cerebrovascular accident, and renal failure. Currently, 1,485,862 patients are enrolled nationwide in the CHP and near 400,000 patients in the metropolitan area of Santiago.

The objectives of this study were twofold. First, to evaluate the proportion of CVRF in hypertensive patients, with or without diabetes, followed under the CHP and, second, to assess the efficacy of this program in the blood pressure control on this population.

## 2. Methods

### 2.1. Population Sample and Procedures

A multicenter cross-sectional systematically randomized sample from a universe of 316,654 hypertensive patients followed throughout the CHP in 133 primary care centers was obtained from 52 municipalities of the metropolitan region of Santiago, Chile. Patients who had BP readings ≥140/90 mmHg throughout their routine medical followups were referred to the CHP. Primary care centers with >1,000 hypertensive patients (97 from 133 centers) were randomly selected, one per each of the 52 municipalities. A randomly selected sample of 1,194 hypertensive patients representing proportionally the total number of patients followed in each one of the primary care centers selected was included in the study. The sampling size (*n* = 1,194) was obtained with 95% of confidence, estimated error of 5%, expected prevalence of BP < 140/90 mmHg of 50%, and design effect of 3.0. Patients were given follow-up appointments according to the clinical assessment and a minimum of three visits per year after the initial evaluation. Patients under 20 years of age, or suffering significant disabilities (bedridden, mentally incompetent, wheel chair users), or with missing appointments (less than three followup visits per year) were excluded (*n* = 23). After replacing the exclusions according to a random procedure using the EPI-Info software, each patient was randomly selected in every one of the primary care centers included.

The data collection was completed in November 2006. The medical records of 12 consecutive months of followup were reviewed. Systolic and diastolic blood pressure (SBP and DBP, resp.) measured by trained personnel throughout 3 successive controls at the corresponding CHP primary care center according to international guidelines [5] and using mercury sphygmomanometers was included for data analysis. Occasional BP measurements performed outside the CHP were not considered. Age, gender, weight, height, education level (years of schooling), and smoking habit (daily smokers) were recorded. Presence of diabetes was established by clinical diagnosis and/or therapies prescribed. All the antihypertensive medications were recorded. Cholesterol levels (total and HDL) were obtained by standard techniques and included for data analysis and only the last ones recorded throughout the follow-up period.

All patients received lifestyle changes counseling (diet, smoking cessation, physical activities) and antihypertensive therapy freely prescribed and adjusted by the physician during the follow-up visits in every primary care center.

### 2.2. Statistical Analysis

The demographic distribution by gender and age of the 316,654 patients was used to calculate the base weight for each sampling unit. Absolute expansion sample weights were calculated using the respective geographical distribution of each one of the primary care centers included in the study allowing to minimize selection bias. Internal and external validity was established by the *Z*-binomial test comparing the sample (1,194) and the universe (316,654). A multivariate logistical analysis was performed to calculate the odds ratio (OR) 95% confidence intervals for the proportion of CVFR (high blood pressure, total cholesterol, HDL cholesterol, body mass index (BMI), smoking, education level) in hypertensive diabetic and nondiabetic patients. In addition, the association between the control of BP and different factors was assessed in age- and sex-adjusted logistic regression models.

## 3. Results

The age distribution showed no statistically significant differences between the universe and the sample. Mean age was 63.7 ± 13.6 years, with no differences between men and women (64.5 ± 13.1 versus 63.7 ± 13.6 years, *P* = 0.13) (Table 1). A small proportion of patients with stable cardiovascular comorbidities under routine specialized care were observed in the sample: ischemic heart disease, 1.5%; congestive heart failure, 3.8%; cerebrovascular accident, 3.2%; renal failure, 1.9%; arrhythmias, 1.6%.

More hypertensive women than men were captured by the CHP (ratio 2.1 : 1).  Table 2 includes the CVRF values (%) in this population. Women had a higher proportion of obesity than men (47.9% versus 33.1%, *P* < 0.01) and HDL < 50 mg/dL in 51.8%; in contrast, men had HDL < 40 mg/dL in 33.2% (*P* < 0.01). Men exhibited higher proportion of smoking, overweight (BMI > 25–30 kg/m^2^), and diabetes than women (*P* < 0.01).  Table 3 compares the CVRF expressed as percentage found in this study with the results reported by the 2010 Chilean National Health Survey [11] in hypertensive and normal individuals.

The mean SBP was 135 ± 15 mmHg, and it was higher in men than in women (138 ± 16 versus 135 ± 15 mmHg, resp., *P* < 0.01). The mean DBP was 81 ± 10 mmHg, with no statistically significant differences by gender. It is of note that 59.7% of the patients achieved a BP < 140/90 mmHg, although women had a larger proportion of controlled BP than men (63.7% versus 52.4%, *P* < 0.01).

**Figure 1:**
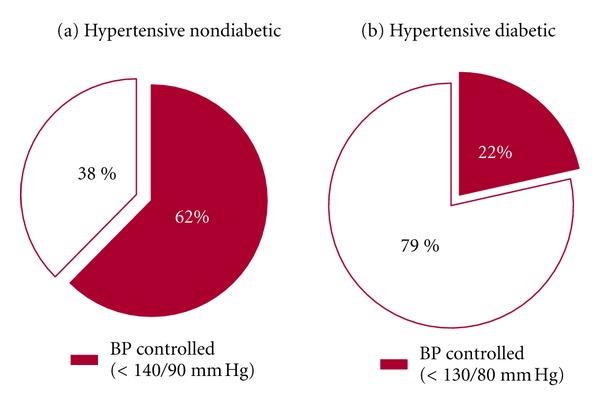
Proportion of satisfactory blood pressure (BP) control in the hypertensive population followed through the Cardiovascular Health Program according to diabetic status.

There were no statistically significant age differences between diabetic and nondiabetic hypertensive patients (64.0 ± 12.2 versus 62.9 ± 14.0 years;  *P* = 0.18). Hypertensive diabetic patients had worse BP control than nondiabetics, achieving a BP < 140/90 mmHg in 53.2% versus 62.4%, respectively (*P* < 0.01), difference that persists although attenuated when considering a level of control <130/80 mmHg (21.5% versus 24.9%, *P* < 0.01) (Table 4, Figure 1). Hypertensive diabetic patients also had a higher proportion of obesity (BMI > 30 kg/m^2^) and low HDL (<40 mg/dL) than nondiabetics (Table 4). After adjusting by age and gender, diabetes and low education level were associated with BP ≥ 140/90 mmHg (OR 1.39 and 1.29, resp., Table 5) and, to a lesser extent, total cholesterol, low HDL cholesterol, and BMI > 30 kg/m^2^. Coexisting incidental cardiovascular diseases were associated to BP < 140/90 mmHg (OR 0.77).

**Figure 2:**
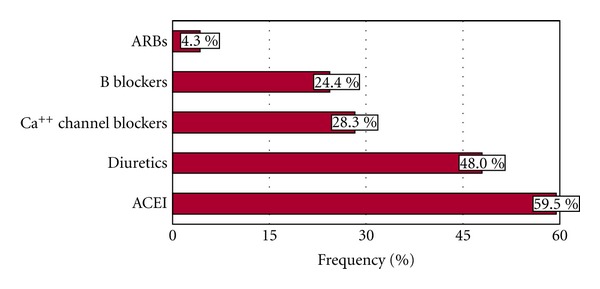
Antihypertensive drugs utilized in the Cardiovascular Health Program (CHP).

**Figure 3:**
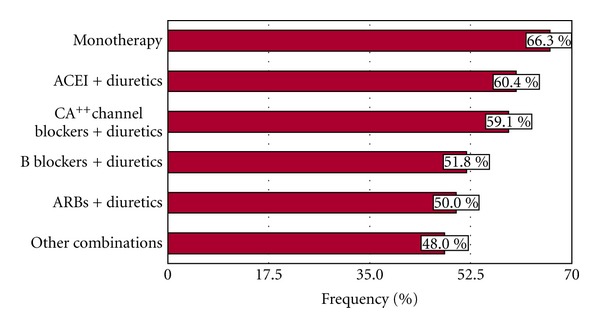
Antihypertensive drug therapy modalities used to achieve BP control (<140/90 mmHg) in the Cardiovascular Health Program (CHP).

A large percentage of patients (91.5%) received antihypertensive drug therapy (34.3% monotherapy and 57.1% combination of drugs). Angiotensin-converting enzyme inhibitors (ACEIs), diuretics, calcium channel, and *β*-blockers were the more frequently used drugs (Figure 2). Exclusively nonpharmacological measures were used in 8.5% of patients, and 75% of them achieved a BP < 140/90 mmHg, whereas only 57% of those under multidrug-therapy group reached that goal. The relationship of BP control with the type of therapy and the number of drugs used is shown in Figure 3. As expected, a greater use of a combination of antihypertensive drugs (more often ACEI, angiotensin receptor blockers (ARBs), *β*-blockers and diuretics) occurred in hypertensive patients with BP more difficult to control. Some combinations appeared to be more effective than others (ACEI + diuretic and Calcium channel blocker + diuretic versus *β*-blocker + diuretic and ARB + diuretic) (Figure 3). There were no differences in the drug monotherapy utilized in nondiabetic and diabetic hypertensive patients. ACEI plus diuretics were more commonly used in nondiabetics, and ARBs plus diuretics more often provided to diabetic patients (Table 6).

## 4. Discussion

This study, which included a representative sample of the hypertensive population followed through the Chilean Cardiovascular Health Program (CHP) in the metropolitan region of Santiago (316,654 patients), documented a BP control of <140/90 mmHg in 59.7% of the patients and 91.5% under antihypertensive drug therapy, comparable to the best results obtained elsewhere [12]. In recent data released by the Center of Disease Control in USA based in the National Health and Nutrition Examination Survey (NHANES) only 69% of hypertensive patients were under antihypertensive drug therapy and 46% had their BP < 140/90 mmHg [5]. Furthermore, a significant improvement was documented in reference to previous Chilean National Health Surveys that showed only 45.3% patients reaching that goal [11]. These findings suggest that a primary care system with a program aiming to detect hypertensive patients and provide them with unrestricted access to medical monitoring and a comprehensive treatment program including lifestyle changes counseling can be an effective preventive strategy.

Nowadays, there is agreement that the simple access to medical followup through provision of medical insurance has not shown satisfactory BP control in hypertensive patients [2, 3, 5, 7]. Interestingly 8.5% of patients in our primary care-based cohort were managed exclusively with nonpharmacological measures, and 75% reached a target BP < 140/90 mmHg underscoring the importance of access to ancillary services as dietary and lifestyle changes counseling (e.g., increasing physical activity) along with the medical followup. In contrast, drug combinations were used in more than 50% of the subjects, achieving the desired goal of BP < 140/90 mmHg only in 57% and as expected, less frequently in patients with higher and more difficult BP to control. These results are probably reflecting differences in the severity of hypertension, therapeutic adherence, or other unknown factors in the population studied.

In regard to the factors identified to influence the control of BP, after adjusting for age and gender in this Chilean hypertensive cohort, diabetes and low education level were associated to worse BP control—and, to a lesser extent, obesity and blood lipids. The association between poor control of BP and other CVRF is probably a reflection of the difficulties for simultaneously controlling multiple risk factors, especially in diabetic hypertensive patients. On the other hand, in cross-sectional and prospective cohort studies in different countries—including Chilean adults—an inverse association between education level and CVRF, cardiovascular events and all-cause mortality has been consistently established [13–21]. This study provides additional evidence suggesting that low educational level seems to be a predictor of poor control of BP in Chilean hypertensive patients. Plausible explanations are related to the acquired skills and knowledge for self-care, healthy lifestyles, and better adherence to antihypertensive therapies with increasing education levels [21]. The presence of cardiovascular complications also showed association with controlled BP, perhaps due to a difference in the lifestyle modification counseling and a better adherence to antihypertensive drug therapy after incidental complications.

Several findings in this study represent a significant challenge for the CHP. First, the much larger proportion of women than men (ratio 2.1 : 1) who entered the CHP is not consistent with the Chilean prevalence of hypertension, which is similar in both genders according to previous National Health Surveys [11]. This fact indicates that the program is not providing adequate coverage to the male population. Probably labor-related hurdles in men limit their availability for routine medical followups in contrast to women, most of them homemakers with more flexible time at their disposal. Second, the large proportion of hypertensive diabetic patients with unsatisfactory BP control, 22% reaching the recommended BP < 130/80 mmHg, underscores the need to improve the BP management in this group. In addition, they had a higher proportion of obesity and lower HDL than nondiabetic hypertensive patients making them particularly vulnerable to cardiovascular events. Finally, this study shows that the presence of diabetes in this hypertensive population is significantly higher (26.4%) than the prevalence (5.1%) in the general Chilean population [11]. According to these results, over 100,000 patients treated in the CHP present a comorbidity of hypertension and diabetes in the metropolitan area. It is conceivable that a better management of these patients at the primary care level may have a significant impact in reducing the morbidity, mortality, and costs related to cardiovascular complications in this highly vulnerable group of patients [22]. Others have already reported this problem. A CVRF trends study in nondiabetic and Type 2 diabetic patients followed for 35 years (1970–2005, Framingham Heart Study) showed consistently higher BP in diabetics [23]. In Spain, a study found that only 9.8% of the hypertensive diabetics had a BP at the recommended target of <130/80 mmHg. Some have questioned whether the proposed level of control for diabetic hypertensive patients (BP < 130/80 mmHg) is necessary or achievable from the perspective of public health [24, 25]. Our experience in the CHP confirms the difficulties in reaching that goal. 

Like the pioneering studies from the Framingham cohort [26], a Chilean prospective cohort study (San Francisco Project) has suggested that an adequate BP control may significantly reduce nonfatal cardiovascular events (myocardial infarction, unstable angina, and cerebrovascular accidents) in hypertensive population, estimating 80.3% reduction after 5 years (population attributable risk) [16]. Considering this estimate, the adequate BP control in 59.7% of patients could achieve 47.9% reduction of cardiovascular complications after 5 years of followup in the CHP. When compared to the National Health Survey (45.3% achieving BP < 140/90 mmHg), the CHP could predict an additional 11.5% reduction in cardiovascular events in hypertensive population.

There are several limitations in our study. One of them is that we did not determine drug adherence, which will be the subject of a future study. Poor adherence could have been a factor to explain the worst BP control in diabetics. Recently it has been suggested that in diabetics the buffering effect of hypertension on pain sensitivity previously reported by several studies may be magnified, leading to hyperalgesia and therefore to a reluctance of diabetic hypertensive patients to follow the antihypertensive treatment [27–30]. However, one of the inclusion criteria for this study was that patients regularly attended their medical appointments, which could be an indirect suggestion that many of them adhered to the recommended therapy. This requirement may also partially explain the differences found with the results of the previous National Health Survey based on general population [11]. In addition the number of men included in the study compared to women was significantly smaller, and this may have influenced some of the results. Nevertheless, because the CHP is now a part of the primary care program in Chile, the number of hypertensive patients to be followed in the future is expected to increase along with the proportion of men included.

It was of note that the frequency of smoking was lower in this hypertensive population than the prevalence observed in the general population of the Chilean National Health Survey [11], difference that may be related to the lifestyle modification counseling as a part of the CHP. Other positive lifestyle modifications in this population can include reduced salt consumption and increased physical activity. These findings along with the improvements in achieving a satisfactory BP control underscore the significance of the CHP in the Chilean hypertensive population.

## 5. Conclusions

A Cardiovascular Health Program that provides unrestricted access to comprehensive treatment and followup of hypertensive patients seems to be an effective strategy to address a major cardiovascular preventive goal in a middle-income developing country as Chile.

According to the results of this study and based on projections from previous studies it is expected that by improving BP < 140/90 mmHg to 59.7% as a consequence of the CPH, 47.9% reduction of future nonfatal cardiovascular events might occur in the hypertensive population of this program. Remaining challenges are the need to improve the underrepresentation of men and the management of hypertensive diabetic patients.

## Figures and Tables

**Table 1:** Age and gender distribution. Comparison of the weighted sample (*n* = 1,194) and the total hypertensive population (*n* = 316,654) followed in the Cardiovascular Health Program (CHP), metropolitan area, Santiago, Chile.

Age (years)	Men					Women				
	Sample		Hypertensive population		*P*-value	Sample		Hypertensive population		*P*-value
	*n*	%	*N*	%		*n*	%	*N*	%	
20–34	7	1.9	2,102	2.2	0.68	18	2.3	4,793	2.2	0.91
35–44	22	5.7	6,186	6.3	0.61	64	7.7	18,875	8.7	0.29
45–54	63	16.4	14,546	14.9	0.41	147	18.1	41,567	19.2	0.31
55–64	85	22.4	24,465	25.0	0.24	203	25.0	54,626	25.3	0.93
65 or more	204	53.6	50,262	51.4	0.38	381	46.9	96,232	44.5	0.20

Total	381	100	97,561	100		813	100	216,093	100	

**Table 2:** Percentage of cardiovascular risk factors by gender (weighted sample).

	Men *n* = 381	Women *n* = 813	Total *n* = 1,194
Average age (years)	63.7 ± 13.6	64.5 ± 13.1	63.3 ± 13.6
	***Percentage % (CI 95%)***		
Diabetes mellitus (clinical diagnosis)	27.5 (27.2–27.8)	25.9 (25.7–26.1)*	26.4 (26.2–26.5)
Glycemia ≥100 mg/dL	48.9 (48.5–49.1)	42.2 (41.9–42.4)*	44.3 (44.1–44.5)
Overweight BMI > 25–29 Kg/m^2^	47.1 (46.8–47.4)	35.5 (35.3–35.6)*	39.4 (36.6–42.2)
Obesity BMI ≥ 30 Kg/m^2^	33.1 (32.8–33.4)	47.9 (47.7–48.1)*	42.8 (42.2–43.4)
Total cholesterol 200–239 mg/dL	36.6 (36.3–36.9)	34.8 (34.6–35.0)*	35.4 (35.2–35.5)
Total cholesterol ≥240 mg/dL	16.1 (15.8–16.3)	26.1 (25.9–26.3)*	23.0 (22.8–23.1)
Cholesterol HDL <40 mg/dL	33.2 (32.8–33.5)	—	—
Cholesterol HDL <50 mg/dL	—	51.8 (51.6–52.0)*	—
Smoking	21.2 (20.9–21.5)	16.5 (16.3–16.7)*	18.0 (17.9–18.1)
Low education <8 years	56.1 (55.8–56.5)	60.1 (59.8–60.4)*	58.8 (58.6–59.1)

*P < 0.01 for comparison with men.

**Table 3:** Proportion of cardiovascular risk factors in 1,194 hypertensive patients (CHP). Comparison with hypertensive and normotensive individuals from the 2010 National Health Survey (NHS).

	Hypertensive CHP *n* = 1,194	Hypertensive NHS 2010 *n* = 1,101	Normotensive NHS 2010 *n* = 3,775
Average age (years)	63.3 ± 13.6	62.5 ± 13.9	40.1 ± 16.4
	***Percentage % (CI 95%)***		
Diabetes mellitus	26.4 (24.0–29.0)	17.5 (15.3–19.7)*	3.2 (2.6–3.7)^†^
Glycemia (≥100 mg/dL)	44.3 (41.5–47.1)	55.7 (50.2–61.2)*	29.6 (28.1–31.1)^†^
Smoking	18.0 (15.8–20.2)	19.1 (16.8–21.4)	40.2 (38.6–41.8)^†^
Overweight (BMI 25.0–29.9 Kg/m^2^)	39.4 (36.6–42.1)	35.8 (32.9–38.6)	36.4 (34.9–37.9)
Obesity (BMI ≥ 30 Kg/m^2^)	42.8 (40.0–45.6)	44.4 (41.5–47.3)*	23.5 (22.1–24.9)^†^
Total cholesterol (≥200 mg/dL)	35.4 (32.5–38.2)	37.2 (34.3–40.1)	53.1 (51.5–54.7)^†^
Low education < 8 years	58.8 (55.6–62.8)	27.9 (25.3–30.5)*	47.5 (45.9–49.0)^†^

*P < 0.01  for comparison with CHP sample,  ^†^ *P* < 0.01 for comparison with CHP sample.

**Table 4:** Cardiovascular risk factors: comparison between hypertensive diabetic and nondiabetic patients.

Risk factors	Hypertensive nondiabetic *n* = 882	Hypertensive diabetic *n* = 312	OR (IC 95%) *n* = 1,194
Average age (years)	62.9 ± 14.0	64.0 ± 12.2	—
	***Percentage % (CI 95%)***		
Blood pressure ≥ 140/90 mmHg	38.0 (34.7–41.2)	46.8 (41.3–52.3)*	1.41 (1.08–1.84)
Blood pressure ≥ 130/80 mmHg	75.1 (72.2–77.9)	78.5 (73.9–83.0)*	1.19 (0.87–1.62)
Total cholesterol 200–239 mg/dL	36.5 (33.1–39.8)	32.6 (27.1–38.0)*	0.84 (0.63–1.12)
Total cholesterol ≥ 240 mg/dL	23.9 (21.0–26.9)	20.7 (15.9–25.4)*	0.83 (0.60–1.17)
HDL cholesterol < 40 mg/dL	19.3 (16.5–22.0)	32.4 (26.6–37.5)*	2.03 (1.45–2.85)
Overweight (BMI 25.0– 29.0 Kg/m^2^)	40.7 (37.5–43.9)	35.3 (30.0–40.6)*	0.78 (0.59–1.01)
Obesity (BMI ≥ 30 Kg/m^2^)	39.5 (36.3–42.7)	51.9 (46.3–57.4)*	1.79 (1.37–2.38)
Low education (<8 years)^†^	39.3 (35.1–43.5)	44.5 (37.8–51.2)*	1.34 (1.32–1.38)
Smoking^††^	18.3 (15.7–20.8)	16.1 (12.0–20.2)*	1.51 (1.47–1.55)

OR refers to odds ratio adjusted by age and gender; the reference group is hypertensive nondiabetic; ^†^the category for nonresponse (38%) is included in multivariate analyses; ^††^OR estimated for the category of daily smoker, including the category for nonresponse in multivariate analyses; *P < 0.01 for comparison with nondiabetic hypertensive patients.

**Table 5:** Association of different factors with the control of blood pressure (BP) in a cohort of hypertensive patients, Cardiovascular Health Program (CHP), Chile.

Risk factors	BP < 140/90 mmHg *n* = 713	BP ≥ 140/90 mmHg *n* = 481	OR (IC95%) *n* = 1,194
Average age (years)	61.9 ± 13.5	65.3 ± 13.4	—
	***Percentage % (CI 95%)***		
Diabetes mellitus	23.3 (20.2–26.4)	30.4 (26.3–34.5)	1.39 (1.37–1.41)
Total cholesterol 200–239 mg/dL	30.4 (27.0–33.8)	34.1 (29.9–38.3)	1.18 (1.16–1.20)
Total cholesterol > 240 mg/dL	20.9 (17.9–23.9)	20.6 (17.0–24.2)	1.02 (1.01–1.04)
HDL cholesterol < 40 mg/dL	21.6 (18.6–24.6)	25.2 (21.3–29.1)	1.17 (1.15–1.20)
Overweight (BMI 25.0–29.0 Kg/m^2^)	38.3 (34.7–41.9)	40.7 (36.3–45.1)	1.03 (1.01–1.04)
Obesity (BMI > 30 Kg/m^2^)	42.4 (38.8–46.0)	43.2 (38.8–47.6)	1.17 (1.15–1.18)
Low education (<8 years)^†^	22.6 (19.5–25.7)	28.5 (24.5–32.5)	1.29 (1.27–1.38)
Smoking	19.6 (16.7–22.5)	14.8 (11.6–18.0)	0.97 (0.95–0.99)
Presence cardiovascular deiseases^Ψ^	8.9 (6.8–11.0)	8.3 (5.8–10.8)	0.77 (0.75–0.79)

OR refers to odds ratio adjusted by age and gender; the group of reference is BP < 140/90; ^†^the category for nonresponse (38%) is included in multivariate analyses; *P < 0.01 for comparison with BP controlled, ^Ψ^presence of stroke, heart failure, and ischemic heart disease.

**Table 6:** Comparison of antihypertensive treatment modalities in hypertensive diabetic and nondiabetic patients.

Treatment modalities	Hypertensive nondiabetic patients *n* = 882 (%)	Hypertensive diabetic patients *n* = 312 (%)	*P*-value
Nonpharmacological	9.0	7.1	0.298
Pharmacological	91.0	92.9	0.298
Monotherapy	34.2	34.6	0.904
ACEI + diuretic	20.7	15.1	0.028
Calcium channel blockers + diuretic	6.8	6.1	0.663
*β*-blockers + diuretic	2.5	2.4	0.804
ARBs + diuretic	0.1	1.6	0.006
Other drugs combination	24.5	29.8	0.065
